# NRF2/Itaconate Axis Regulates Metabolism and Inflammatory Properties of T Cells in Children with JIA

**DOI:** 10.3390/antiox11122426

**Published:** 2022-12-08

**Authors:** Anandhi Rajendiran, Sudheendra Hebbar Subramanyam, Patricia Klemm, Vera Jankowski, Jorg van Loosdregt, Bas Vastert, Kristina Vollbach, Norbert Wagner, Klaus Tenbrock, Kim Ohl

**Affiliations:** 1 Department of Pediatrics, Pediatric Rheumatology, Medical Faculty, RWTH Aachen, 52074 Aachen, Germany; 2 Institute for Molecular Cardiovascular Research, RWTH Aachen University Hospital, 52074 Aachen, Germany; 3 Laboratory for Translational Medicine, Department of Pediatric Immunology & Rheumatology, University Medical Centre Utrecht, 3584 Utrecht, The Netherlands

**Keywords:** NRF2, JIA, redox metabolism, ROS, immunometabolism

## Abstract

Background: CD4+ T cells critically contribute to the initiation and perturbation of inflammation. When CD4+ T cells enter inflamed tissues, they adapt to hypoxia and oxidative stress conditions, and to a reduction in nutrients. We aimed to investigate how this distinct environment regulates T cell responses within the inflamed joints of patients with childhood rheumatism (JIA) by analyzing the behavior of NRF2—the key regulator of the anti-oxidative stress response—and its signaling pathways. Methods: Flow cytometry and quantitative RT-PCR were used to perform metabolic profiling of T cells and to measure the production of inflammatory cytokines. Loss of function analyses were carried out by means of siRNA transfection experiments. NRF2 activation was induced by treatment with 4-octyl-Itaconate (4-OI). Results: Flow cytometry analyses revealed a high metabolic status in CD4+ T cells taken from synovial fluid (SF) with greater mitochondrial mass, and increased glucose and fatty acid uptake. This resulted in a heightened oxidative status of SF CD4+ T cells. Despite raised ROS levels, expression of NRF2 and its target gene *NQO1* were lower in CD4+ T cells from SF than in those from blood. Indeed, NRF2 activation of CD4+ T cells downregulated oxidative stress markers, altered the metabolic phenotype and reduced secretion of IFN-γ. Conclusion: NRF2 could be a potential regulator in CD4+ T cells during chronic inflammation and could instigate a drift toward disease progression or regression, depending on the inflammatory environment.

## 1. Introduction

Distinct T cell subsets use different energy and biosynthetic pathways to fulfill their energy demands or sustain their activities [1,2]. In addition, some adaptations of T cellular metabolisms are disease related. T cells from systemic lupus erythematosus (SLE) patients display a chronically activated phenotype with enhanced citrate cycle activity and dependency on oxidative phosphorylation (OXPHOS) to fulfill their energy needs [3]. Naïve T cells from rheumatoid arthritis patients (RA) shunt glucose away from glycolysis towards the pentose phosphate pathway (PPP) after activation [4] and favor lipogenesis over lipolysis [5]. The outcome is a reductive cellular environment in which fatty acids are stored in lipid droplets and utilized for the building of invasive membrane ruffles. Subsequently, T cells become hyper-proliferative and prone to invade tissue [6]. This suggests that metabolic reprogramming is not only important for supplying cells with energy and biosynthetic precursors but that it also directly regulates their functions and activities.

Metabolic programming is partly regulated by metabolites [7], which control cell fate and function by regulating chromatin modifications, DNA methylation and post-translational modifications [8,9]. Metabolites can thereby alter T cell responses. For example, citrate exported from the mitochondria supports epigenetic remodeling in activated T cells and is required for differentiation [10,11]. Itaconate is a metabolite known to play a central role in activated macrophages by linking oxidative stress responses, cell metabolism and immune responses [12], Itaconate is produced in macrophages in response to LPS, and promotes anti-inflammatory programs through Succinate dehydrogenase (SDH) [13,14] and also through nuclear factor (erythroid-derived 2)-like 2 (NRF2) activation [15]. Activation of NRF2 occurs by the alkylation of the key redox-sensing protein KEAP1. NRF2 expression is known to be increased in activated T cells in mice and humans [16,17,18], although the exact role of NRF2 in T cells remains unclear. Constitutive activation of Nrf2 in T cells was protective in a murine acute kidney injury model and led to higher frequencies but not total numbers of intrarenal regulatory T cells (Tregs), as well as reduced expression of inflammatory cytokines in CD4+ T cells [19]. However, constitutive ablation of Nrf2 in T cells ameliorated graft-vs-host disease (GvHD) [18]. Again, a study with human cells indicates that high levels of NRF2 in CD8+ T-cells may be protective against chronic GvHD [20]. While Tregs have been reported to be more resistant to oxidative stress-induced cell death than conventional T cells [21], other authors have found higher vulnerability of Tregs to free oxygen species, which was attributed to their weak NRF2-associated antioxidant system [22]. This diminished NRF2-associated antioxidant system facilitates Treg apoptosis but nevertheless causes enhanced immunosuppressive capacity within the tumor microenvironment. Our own study indicates that Nrf2 is a negative regulator of murine Treg function and that Foxp3-specific activation of Nrf2 in mice results in a loss of Foxp3 expression and spontaneous inflammation [17].

Although the role of itaconate has been intensively studied in macrophages, much less is known about its function in human T cells. Furthermore, the exact role played by NRF2 in human T cells remains unclear. This is partially due to discrepancies between mice and humans and to the fact that ex vivo analyses can only partly reproduce in vivo situations of chronic inflammation. To fill this gap, we directly analyzed the redox metabolism of T cells within the local inflammatory environment by using T cells derived from inflamed sites. The inflamed joints of JIA patients are infiltrated by mononuclear cells, including T and B lymphocytes, dendritic cells and macrophages [23,24,25]. As in all inflamed tissues, T cells within arthritic joints have to adapt to low levels of oxygen, a lack of key nutrients and oxidative stress conditions [26]. Inflamed tissues are known to have low levels of glucose [27] and glutamine [28] but quite an abundance of fatty acids [29,30]. In this study, we set out to analyze redox metabolism in inflamed tissues by examining the NRF2/itaconate axis in JIA.

## 2. Materials and Methods

### 2.1. Patients and Healthy Donors

All patients enrolled were diagnosed as having oligoarticular JIA and were receiving nonsteroidal anti-inflammatory drugs before therapeutic aspiration of synovial fluid (SF) and administration of corticosteroids. Cells were pelleted by centrifugation and supernatants were stored at −80 °C. Ethical approval for all experiments was obtained from the local ethics committee. All patients provided fully informed consent or age-appropriate assent where applicable (Table 1).

### 2.2. Cell Isolation

Human peripheral blood mononuclear cells (PBMC) from patients with JIA and healthy donors were isolated on a Ficoll (PAN Biotech, Aidenbach-Germany; P04-601000) gradient. SF was obtained by incubation with hyaluronidase for 30 min. followed by centrifugation at 400× *g* for 10 min. The pelleted cells were used to isolate synovial fluid mononuclear cells (SFMC) by Ficoll gradient.

### 2.3. Cell Culture

CD4+ T cells were isolated by magnetic cell separation using CD4 Microbeads, human (Miltenyi Biotec, Bergisch Gladbach-Germany; 130-045-101). PBMCs or CD4+ T cells from healthy donors were cultured in RPMI 1640 medium (ThermoFisher Scientific (Gibco), Karlsruhe, Germany; 21875-034) supplemented with 10% heat-inactivated FBS (ThermoFisher Scientific (Gibco), Germany; 10500-064) and 1% penicillin/streptomycin (ThermoFisher Scientific (Gibco), Germany; 15140-122) and incubated with 10% allogenic SF or serum from healthy controls (HC) for 18 h. For proliferation assays, the CD4+ T cells were labeled with cell proliferation dye eFluor670 (5 µM) (ThermoFisher Scientific, Germany; 65-0840-85) according to the manufacturer’s instructions. The CD4+ T cells were stimulated with 5 µg/mL anti-CD3(OKT3) and 1 µg/mL anti-CD28(CD28.2) (ThermoFisher Scientific, Germany; 16-0037-85 and 16-0289-85, respectively); the cells were treated with 20 µM 4-OI or (DMSO) as a vehicle control for 72 h.

### 2.4. Flow Cytometry

For surface staining, single-cell suspensions were stained with anti-CD4 (clone OKT-4) Pacific Blue (PB) labeled antibody (1:100, ThermoFisher Scientific, Germany; 48-0048-42). To analyze NRF2 and NQO1, cells were fixed and permeabilized with a FOXP3/Transcription factor staining buffer set (ThermoFisher Scientific, Germany; 00-5523-00) following the manufacturer’s instructions. The cells were stained first with unconjugated polyclonal NRF2 antibody (1:50, Rabbit anti-NRF2, GeneTex, Irvine, CA, USA; GTX103322), unconjugated polyclonal NQO1 antibody (1:110, Rabbit anti-NQO1, ThermoFisher Scientific, Germany PA5-21290) and unconjugated monoclonal isotype control antibody (1:220, Rabbit IgG [EPR25A], Abcam, Berlin, Germany; ab172730), and then with FITC labeled Goat anti-Rabbit IgG (1:200, BD Biosciences, Heidelberg-Germany; 554020). Fatty acid uptake was measured by BODIPY FL C12 (ThermoFisher Scientific, Germany; D3822) on FITC at a concentration of 2 µM and Lipid content was measured using BODIPY 493/503 (ThermoFisher Scientific, Germany; D3922) on FITC at a concentration of 2 µM by following the manufacturer’s instructions. Measurement of ROS was performed by means of Total Reactive Oxygen Species (ROS) Assay Kit on FITC (ThermoFisher Scientific, Germany; 88-5930-74) and mitochondrial ROS by 5 µM MitoSoxRed on PE (ThermoFisher Scientific, Germany; M36008) by following the manufacturer’s instructions. Mitochondrial mass measurements were performed with 500 nM MitoTracker on FITC (ThermoFisher Scientific, Germany; M7514). 2-deoxy-2-[(7-nitro-2,1,3-benzoxadiazol-4-yl)amino]-D-glucose (2-NBDG) was measured by means of a glucose uptake cell-based kit at a concentration of 125 µg/mL on FITC (Cayman Chemical, Ann Arbor, MI, USA; 600470) by following the manufacturer’s instructions. To analyze IFN-γ the cells were stimulated with 0.5 µg/mL PMA (Merck, Darmstadt-Germany; P8139), 1 µg/mL Ionomycin (Merck; I0634) and 1 µL/mL Golgi plug (BD Biosciences, Heidelberg-Germany; 555029) for 5 h at 37 °C and thereafter fixed and permeabilized with a FOXP3/Transcription factor staining buffer set. Then the cells were stained with anti- IFN-γ (clone 4S.B3) on APC labeled antibody (1:100, ThermoFisher Scientific, Germany; 17-7319-82). Flow cytometry was carried out using the FACSCanto II device (BD Biosciences, Heidelberg-Germany). Data analysis was performed using FCS Express software. The gating strategies are shown in Supplementary Figure S1.

RNA isolation, complementary DNA (cDNA) synthesis, and quantitative real-time polymerase chain reaction (RT-qPCR).

Total RNA was extracted from cells using an RNeasy Plus Mini Kit (Qiagen, Hilden-Germany; 74136) and transcribed to cDNA using RevertAid H Minus First Strand cDNA Synthesis Kit (ThermoFisher Scientific, Germany; K1631) according to the manufacturer’s instructions. Standard RT-qPCR was carried out on a TaqMan 7900 (ThermoFisher Scientific (Applied Biosystems), Waltham, MA, USA) using the DNA intercalating dye SYBR Green. Each measurement was set up in duplicate, after normalization to endogenous housekeeping control gene *ACTB* (Actin Beta), and the relative expression was then calculated. The following primers were used:
*ACTB*For 5′ AGA TGG CCA CGG CTG CT 3′
Rev 5′ AAC CGC TCA TTG CCA ATG G 3′*IL17a*For 5′ GAATCTCCACCGCAATGAGGA 3′
Rev 5′ TGGTAGTCCACGTTCCCATCAG 3‘*NRF2*For 5′ TCC AGT CAG AAA CCA GTG GAT 3′
Rev 5′ AAT GTC TGC GCC AAA AGC TG 3′*NQO1*For 5′ GGT TTG AGC GAG TGT TCA TAG G 3′
Rev 5′ GCA GAG AGT ACA TGG AGA CAC 3′*HO-1*For 5′ CAG TGC CAC CAA GTT CAA GC 3′
Rev 5′ GTT GAG CAG GAA CGC AGT CTT 3′*TRX1*For 5′ CCC TTT CTT TCA TTC CCT CTC TG 3′
Rev 5′ ATT CAC CCA CCT TTT GTC CCT 3′*IFN-γ*For 5′ ACT AGG CAG CCA ACC TAA GCA AGA 3′
Rev 5′ CAT CAG GGT CAC CTG ACA CAT TCA 3′

### 2.5. Transfection of Primary Cells

Briefly, 2.5 × 10^6^ cells were suspended in 100 µL of T-cell suspension buffer. To this 10 nM *NFE2L2* siRNA (Ambion, Frankfurt, Germany; catalog No. 4392420) or a control siRNA (Ambion, Frankfurt, Germany; catalog No. 4390843) was added and the transfection was carried out according to the manufacturer’s instructions (Neon transfection, ThermoFisher Scientific; MPK10025). After the transfection, the transfected cells were added to the 1.9 mL of RPMI + 10%FCS (without antibiotics) in a 12-well culture plate and incubated for 24 h at 37 °C. After 24 h of incubation, the transfected cells were stimulated in a 96-well U-bottom plate with anti-CD3 (5 µg/mL) and anti-CD28 (1 µg/mL) (both from ThermoFisher Scientific) for 24 h. For 4-OI experiments, the same protocol was followed. However, after the first 24 h incubation of transfected cells, the cells were cultured in the presence of anti-CD3 and anti-CD28, with 20 µM 4-OI or with vehicle (DMSO) as a control and incubated for 48 h. After the final incubation, the cells were used for further analysis (flow cytometry and RT-qPCR).

### 2.6. SDS Page and Western Blot

40 × 10^6^ PBMCs per condition were incubated for 4 h with 10 % HC or SF. Nuclear and cytosolic protein extraction was performed according to the nuclear fractionation protocol provided by Abcam. Therefore, PBMCs were lysed for 10 min on ice in buffer A (10 mM HEPES, 1.5 mM MgCl_2_, 10 mM KCl, 0.05 & NP-40, 1x Proteinase Inhibitor Cocktail), centrifuged for 10 min at 4 °C at 3000 rpm and the supernatant containing the cytosolic protein extract removed and stored at −20 °C until further analysis. The pellet was resuspended in 300 µL buffer B (5 mM HEPES, 1.5 mM MgCl2, 0.5 mM EDTA, 26% glycerol, 300 mM NaCl) and homogenized using sonification. Afterward, the samples were incubated for 30 min on ice, centrifuged for 20 min at 4 °C at 17,000× *g* and the supernatant containing the nuclear protein extracts was stored at −20 °C until further analysis. 15 × 10^6^ PBMCs transfected with NFE2L2 siRNA or control siRNA were incubated for 24 h and total protein extraction was performed using RIPA buffer (Abcam, Berlin, Germany; ab156034) with 1× Proteinase Inhibitor cocktail (Roche, Germany; 11836153001) by incubating on ice for 30 minutes. The cells were centrifuged for 15 min, 14000× *g* at 4°C and the supernatant containing protein extract was collected and stored at −20 °C until further analysis. Protein concentrations were determined with a bicinchoninic acid (BCA) assay (Pierce BCA Protein Assay Kit, ThermoFisher Scientific, Germany; 23225); 40 µg of protein extract was separated on a 10% acrylamide gel at 100 V for 90 min and semi-dry blotted for 60 min at 200 mA onto a nitrocellulose membrane using a Trans-Blot Turbo Transfer System (Bio-Rad, Feldkirchen, Germany). Afterward, the blot was washed, blocked for 60 min at room temperature in 5% BSA, washed again and incubated overnight at 4 °C in primary antibody ((1:500, Rabbit anti-NRF2, GeneTex, Irvine, CA, USA; GTX103322) (1:100, Mouse anti-LaminA/C, Santa Cruz, TX, USA; sc-376248)). The next day the blot was washed again and incubated for 60 min at room temperature in horseradish peroxidase (HRP)-conjugated secondary antibody (1:5000; polyclonal goat anti-rabbit HRP, Santa Cruz, TX, USA; sc-2004, polyclonal goat anti-mouse HRP, Santa Cruz, TX, USA; sc-2005), washed again and HRP signal was detected on a ChemiDoc MP Imaging System (Bio-Rad, Feldkirchen, Germany) or LAS-3000 (Fujifilm, Louisa, VA, USA) using the SuperSignal West Dura Extended Duration Substrate (ThermoFisher Scientific, Germany; 34075). Protein expression was quantified using the ImageJ software (NIH).

### 2.7. Mass Spectrometry

15 × 10^6^ CD4+ T cells from healthy controls were treated with 10% HC/SF serum and incubated for 18 h. Total protein extraction was conducted using RIPA buffer with a 1× protease inhibitor cocktail. 40 µg of protein extract was separated on a 10% acrylamide gel at 100 V for 90 min. Samples were digested with 0.02 μg/μL trypsin and ammonium bicarbonate for 24 h at 37 °C. The peptides were then desalted and concentrated by ZipTipC18 technology (Millipore, Billerica, MA, USA) with double-distilled water containing 80% acetonitrile and 0.1% trifluoroacetic acid. The eluate was spread onto a matrix-assisted laser desorption/ionization (MALDI) target plate (MTP AnchorChip 400/384 target plate, Bruker Daltonic, Bremen, Germany) with α-cyano-4-hydroxycinnamic acid used as the matrix. Subsequently a MALDI–time of flight (TOF)/TOF MS (Ultraflex III; Bruker Daltonic) MS was performed.

A database search (Swiss-Prot) using the Mascot 2.2 search engine (Matrix Science Inc., Boston, MA, USA) and Bruker Bio-Tool 3.2 software (Bruker Daltonics, Bremen, Germany) was performed with the calibrated and annotated spectra to calculate the peptide mass signal for each entry into the sequence database, to compare the experimental MALDI-MS and MALDI-MS/MS dataset, and to assign a statistical weight to each individual peptide match using empirically determined factors. Adapted/rewritten from [31].

### 2.8. ChIP Sequencing

ChIP Sequencing was performed as described previously [32]. In short, healthy control PBMCs, and JIA SFMCs, were FACS-sorted for CD4+ CD45RO+ cells and crosslinked with 2% formaldehyde. Crosslinking was stopped by adding 0.2 M glycine and isolated nuclei were lysed in 20 mM Tris (pH 7.5), 150 mM NaCl, 2 mM EDTA, 1% NP-40, 0.3% SDS. Lysates were sheared (Covaris duty cycle 20%, intensity 3, 200 cycles per burst, 60-s cycle time, eight cycles) and diluted in 20 mM Tris (pH 8.0), 150 mM NaCl, 2 mM EDTA, 1% X-100. Sheared DNA was incubated overnight with anti-histone H3 acetyl K27 antibody (Abcam, Boston, MA, USA; ab4729) pre-coupled to protein A/G magnetic beads. Cells were washed and crosslinking was reversed by adding 1% SDS, 100 mM NaHCO3, 200 mM NaCl, and 300 mg/mL proteinase K. DNA was purified using ChIP DNA Clean & Concentrator kit (Zymo Research), end-repair, a-tailing, and ligation of sequence adaptors was conducted using Truseq nano DNA sample preparation kit (Illumina, San Diego, CA, USA). Samples were amplified by PCR, selected for the proper size range and the absence of adaptor dimers and barcoded libraries were sequenced 75 bp single-end on Illumina NextSeq500 sequencer (Utrecht DNA sequencing facility).

### 2.9. Statistics

Differences between the two groups were evaluated using a two-tailed unpaired *t*-test or paired *t*-test if data were normally distributed and the Mann–Whitney test or Wilcoxon signed-rank test if they were non-parametric. To determine the significance of N-fold regulations we performed one sample *t*-test for parametric data and one sample Wilcoxon test for non-parametric data. To compare more than two groups we used ANOVA multiple comparison tests. All statistical analysis and subsequent graphics generation were performed using GraphPad Prism (GraphPad Software, San Diego, CA, USA). Standard deviation (SD) is used for error bars.

## 3. Results

### 3.1. Redox Metabolism Is Dysregulated in JIA T Cells within Inflamed Joints

**Figure 1:**
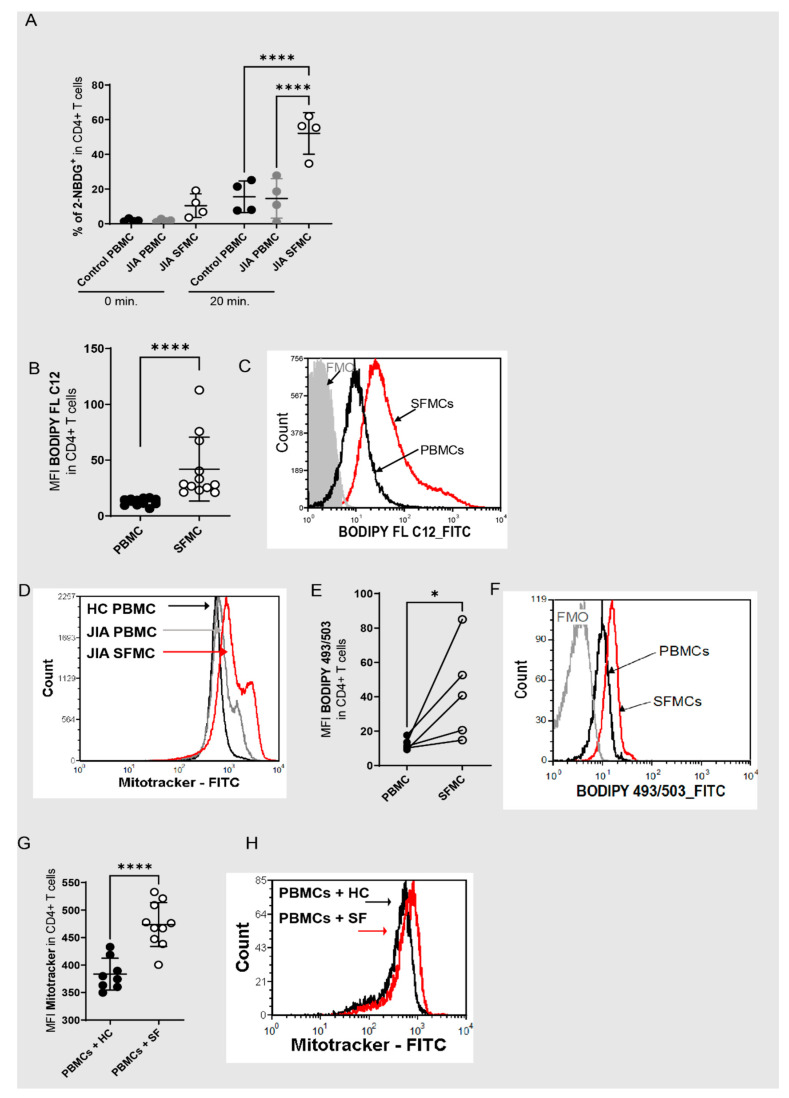
Redox metabolism is dysregulated in T cells within inflamed joints. (**A**) 2-NBDG incorporation was analyzed in CD4+ T cells from healthy control PBMCs (HC PBMCs), JIA PBMCs and SFMCs from the inflamed joints of JIA patients (JIA SFMCs) at different time points (N = 4 for donors and four individual experiments), an ordinary one-way ANOVA Tukey’s multiple comparison test was performed. (**B**) Quantitative results of geometric mean fluorescent intensity (MFI) of BODIPY FL C12 (N = 12 for donors and 12 individual experiments) on JIA PBMCs and SFMCs, and a Mann–Whitney test was performed. (**C**) Representative histogram of the MFI of BODIPY FL C12 in CD4+ T cells from JIA PBMCs (black) and SFMCs (red). (**D**) Representative histogram of the MFI of Mitotracker in CD4+ T cells from HC PBMCs (black), JIA PBMCs (grey) and SFMCs (red). (**E**) Lipid content in CD4+ PBMCs and SFMCs (N = 5 for donors and five individual experiments) measured as BODIPY TM 493/503 MFI, a two-tailed unpaired *t*-test was performed. (**F**) Representative histogram of (**E**) BODIPY TM 493/503 in CD4+ T cells from JIA PBMCs (black) and SFMCs (red). (**G**) MFI of Mitotracker in HC PBMCs after incubation with 10% HC serum (N = 8 for donors and two individual experiments) or 10 % SF (N = 10 for donors and two individual experiments. (**H**) Representative histogram of the MFI of Mitotracker in CD4+ T cells with HC serum (black) and SF serum (red) incubation. Stastical significance represented as * *p* < 0.05, **** *p* < 0.0001.

CD4+ T cells within the joints are highly activated memory T cells [33,34] produce inflammatory cytokines [35,36] and play a dominant role in the inflammatory reaction of the joint. It is, therefore, not surprising that, in comparison to T cells in the peripheral blood, these cells are characterized by high metabolic activity. This is manifested in high uptakes of glucose (Figure 1A) and fatty acids (Figure 1B,C), a greater mitochondrial mass (Figure 1D), and a higher neutral lipid content (Figure 1E,F). In addition, we recently showed higher mitochondrial ROS [37], and a higher overall abundance of ROS in SF T cells [37]. The metabolic and inflammatory activation to which T cells are exposed in inflamed joints can be mimicked in HC T cells by incubation with SF [35,37]. In detail, the effects of SF incubation include a higher secretion of IFN-γ and IL17a, enhanced T cell proliferation, raised mitochondrial ROS as well as higher overall ROS production, and phosphorylation of mTOR [35,37]. In addition, we found a greater mitochondrial mass after incubation of HC T cells with SF (Figure 1G,H). From this, we conclude that redox metabolism is dramatically altered in T cells in inflamed joints and that T cells undergo specific adaptations after their entry into the site of inflammation.

### 3.2. Nrf2 Signaling Is Reduced in SF T Cells

**Figure 2:**
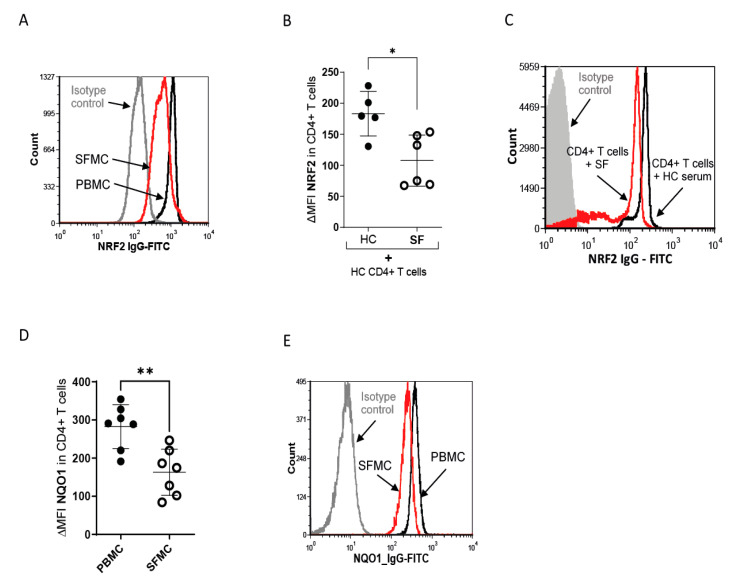
NRF2 protein expression is reduced in SF T cells. (**A**) Representative histogram of NRF2 in SFMCs (red) and PBMCS (black) MFI and IgG isotype MFI (grey). (**B**) Statistical analysis of ΔMFI of NRF2 in HC CD4+ T cells after incubation with 10% HC (N = 5 for donors and two individual experiments) serum or SF (N = 6 for donors and two individual experiments) for 18 h. ΔMFI was calculated as MFI(NRF2)-MFI (IgG) and a two-tailed unpaired *t*-test was performed. (**C**) Representative histogram of NRF2 in HC CD4+ T cells after incubation with SF serum (red) and HC serum (black). (**D**) Statistical analysis of MFI of NQO1 expression in CD4^+^ PBMCs (N = 7 for donors and 6 individual experiments) and SFMCs (N = 7 for donors and six individual experiments); ΔMFI was calculated as MFI(NQO1)-MFI (IgG) and a two-tailed unpaired *t*-test was performed. (**E**) Representative histogram of NQO1 in SFMCs (red) and PBMCS (black) MFI and IgG isotype MFI (grey). Stastical significance represented as * *p* < 0.05, ** *p* < 0.01.

Oxidative stress is associated with JIA and rheumatoid arthritis [38,39]. Furthermore, NRF2 expression is increased in activated T cells in mice and humans (16–18). As SF T cells are activated and exposed to high intra- and extracellular ROS levels, we expected to find higher levels and activity of NRF2 in SF T cells. Surprisingly, NRF2 was downregulated in JIA SF T cells compared to those derived from peripheral blood (Figure 2A). Furthermore, incubation of PBMCs with SF, as opposed to HC serum, resulted in a downregulation of NRF2 expression in T cells, as measured by flow cytometry (Figure 2B,C) and Western blot (Supplementary Figure S2). NQO1, a target gene of NRF2 that is used to determine the activity of the NRF2 pathway [40], was also downregulated in SF T cells (Figure 2D,E).

**Figure 3:**
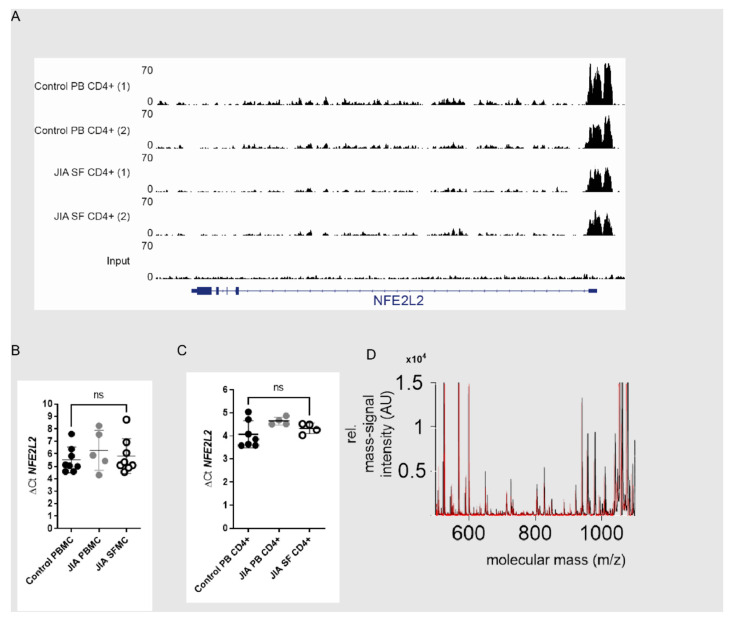
NRF2 is not regulated by transcriptional mechanism and shows no posttranscriptional protein modifications. (**A**) H3K27Ac ChIP-seq tracks at the SLC27A2 loci in T cells from peripheral blood(PB) and synovial fluid (SF). ΔCt is calculated as (Ct (NFE2L2)-Ct (ACTB (housekeeping gene). (**B**) ΔCt of NFE2L2 mRNA expression in HC PBMCs (N = 8 for donors), JIA PBMCs (N = 5 for donors) and JIA SFMCs (N = 8 for donors), an ordinary one-way ANOVA Dunnett’s multiple comparison test was performed. (**C**) ΔCt of NFE2L2 mRNA expression in CD4^+^ T cells from HC peripheral blood (N = 7 for donors and 4 individual experiments), JIA peripheral blood (N = 4 for donors and individual experiments) and synovial fluid from JIA patients (N = 4 for donors and individual experiments), an ordinary one-way ANOVA Dunnett’s multiple comparison test was performed. (**D**) HC CD4^+^ T cells were incubated with either 10% HC (N = 5 for donors and two individual experiments) serum or SF (N = 6 for donors and 2 individual experiments) for 18 h for Mass Spectrometric analysis.

NRF2 activation is mainly regulated at the protein level. Nevertheless, we also checked to see if NRF2 gene transcription is altered in SF T cells. Promoter and enhancer activity within the NFE2L2 locus were studied by analyzing H3K27ac CHIP data from PB T cells and SF T cells (Figure 3A). In line with this, NRF2 (NFE2L2) mRNA expression (Figure 3B,C) also showed no signs of an altered NRF2 transcription in SFMCs or in CD4+ T cells (Figure 3C). We decided to perform mass spectrometry to uncover SF-induced modifications in the NRF2 protein and analyzed five different post-translational modifications (oxidation, phosphorylation, sulfatisation, carbamylation and methylation). We did not find any differences here between T cells incubated with synovial fluid and those incubated with control serum. From this, we concluded that no specific NRF2 protein modifications are induced by SF (Figure 3D). The above findings suggest that the NRF2 pathway is dysregulated in T cells within inflamed joints, either by a post-transcriptional mechanism or by enhanced degradation at the protein level. Given the central role of redox metabolism in inflamed tissue, we next explored the functional effect of this on T cells.

### 3.3. Alteration of Redox Metabolism in T Cells by 4-OI Upregulation of NRF2

**Figure 4:**
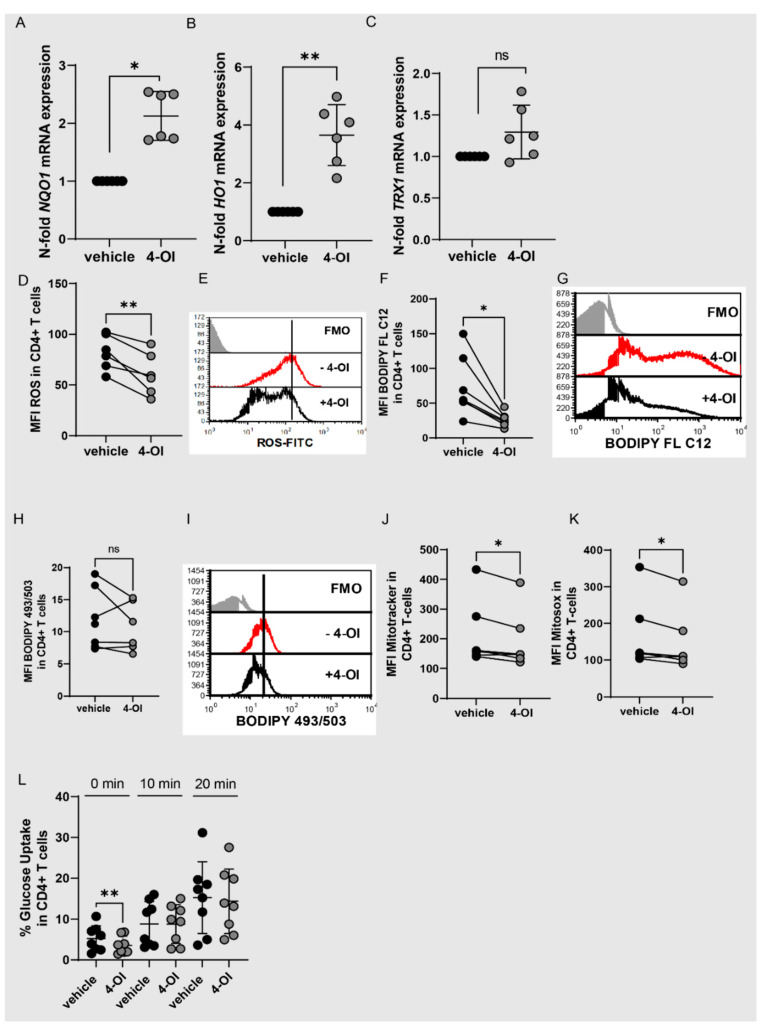
4-OI activates NRF2 while reducing ROS production and fatty acid uptake. (**A**–**C**) CD4^+^ T cells were stimulated with anti-CD3/CD28 for 48 h with 4-OI (20 µM) or vehicle (w/o 4-OI) and analyzed for *NQO1*, *HO1*, *TRX1* expression by RT-qPCR (N = 6 for donors and three individual experiments; SD: 0.4215, 1.052 & 0.3238). A one-sample Wilcoxon test was performed in A) and a one-sample *t*-test in (**B**,**C**). (**D**–**L**) CD4^+^ T cells were stimulated with anti-CD3/CD28 for 48 h with 4-OI (20 µM) or vehicle (w/o 4-OI) and flow cytometric analysis was performed. (**D**) Statistical analysis of MFI of ROS in CD4^+^ T cells (N = 6 for donors and three individual experiments), a two-tailed paired *t*-test was performed. (**E**) Representative histogram showing the geometric mean fluorescent intensity (MFI) of ROS in CD4+ T cells. (**F**) Statistical analysis showing MFI of BODIPY FL C12 to determine fatty acid uptake in CD4^+^ T cells (N = 7 for donors and three individual experiments), a Two-tailed paired *t*-test was performed. (**G**) Representative histogram showing the geometric mean fluorescent intensity (MFI) of BODIPY FL C12 in CD4+ T cells. (**H**) Statistical analysis showing MFI of BODIPY 493/503 to determine lipid content in CD4^+^ T cells (N = 7 for donors and three individual experiments), a Two-tailed paired *t*-test was performed. (**I**) Representative histogram showing the geometric mean fluorescent intensity (MFI) of BODIPY 493/503 in CD4+ T cells. (**J**) Statistical analysis showing MFI of Mitotracker in CD4^+^ T cells (N = 7 for donors and three individual experiments, a Wilcoxon matched-pairs signed rank test was performed. (**K**) Statistical analysis showing MFI of Mitosox in CD4^+^ T cells (N = 7 for donors and three individual experiments), a Two-tailed paired *t*-test was performed. (**L**) Statistical analysis showing percentages of 2-NBDG positive CD4+ T cells to assess glucose uptake (N = 8 for donors and four individual experiments), a two-tailed paired Wilcoxon test was performed. Stastical significance represented as * *p* < 0.05, ** *p* < 0.01 and ns is not significant.

We used 4-octyl itaconate (4-OI) to elucidate the function of NRF2 in T cells. 4-OI is a cell-permeable itaconate derivative (15), which alkylates cysteine residues on KEAP1 and thereby activates NRF2 (15). We used concentrations of 20 µM which did not induce apoptosis and used the same volume of DMSO as vehicle control. As expected, 4-OI upregulated the expression of the NRF2 target genes NQO1, HO1 and TRX1 in T cells (Figure 4A–C). Furthermore, ROS production was significantly reduced (Figure 4D,E). This suggests that 4-OI induces the NRF2 pathway in T cells. We next asked if the NRF2/Itaconate axis alters cell metabolism and might thereby contribute to the adaptation of T cells in the inflamed joints. Indeed, 4-OI did downregulate fatty acid uptake in CD4+ T cells and tended to lower lipid content, but not significantly (Figure 4F–I). It furthermore slightly downregulated mitochondrial mass and mitochondrial ROS (Figure 4J,K). 4-OI did not clearly downregulate glucose uptake (Figure 4L), which suggests that 4-OI specifically targets fatty acid uptake and only slightly affects mitochondrial metabolism while not altering glucose uptake. The metabolic state of T cells is directly linked to their functions.

**Figure 5:**
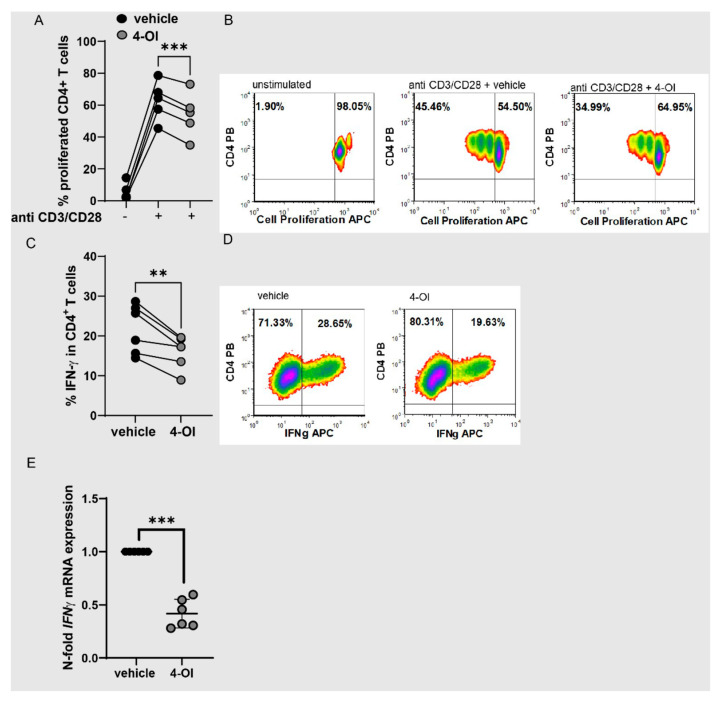
4-OI reduces proliferation and IFN-γ production of CD4^+^ T cells. (**A**,**B**) CD4^+^ T cells were labeled with cell proliferation dye and left unstimulated or stimulated with anti-CD3/CD28 for 72 h with 4-OI or vehicle (DMSO), percentages of proliferated cells were determined by flow cytometry. (**A**) Statistical analysis of proliferated cells, (N = 5 for donors and two individual experiments) a two-tailed paired *t*-test was performed. (**B**) Representative density plots showing percentages of proliferated cells. (**C**,**D**) CD4^+^ T cells were stimulated with anti-CD3/CD28 for 72 h with 4-OI or vehicle (w/o 4-OI) and percentages of IFN-γ+ cells were determined by flow cytometry. (**C**) Statistical analysis of IFN-γ^+^ T cells, (N = 6 for donors and four individual experiments), a two-tailed paired *t*-test was performed. (**D**) Representative density plots of (**C**). (**E**) CD4^+^ T cells were stimulated with anti-CD3/CD28 for 48 h with 4-OI or vehicle (w/o 4-OI) and analyzed for *IFN-γ* expression by RT-qPCR, (N = 6 for donors and three individual experiments; SD: 0.1348), a one sample *t*-test was performed. Stastical significance represented as ** *p* < 0.01, *** *p* < 0.001.

To analyze whether 4-OI also directly regulated T cell functions, we measured CD4+ T cell proliferation and cytokine expression. 4-OI reduced proliferation (Figure 5A,B) and IFN-γ production (Figure 5C–E).

### 3.4. 4-OI Affects T Cell Function in a Nrf2-Dependent Manner

**Figure 6:**
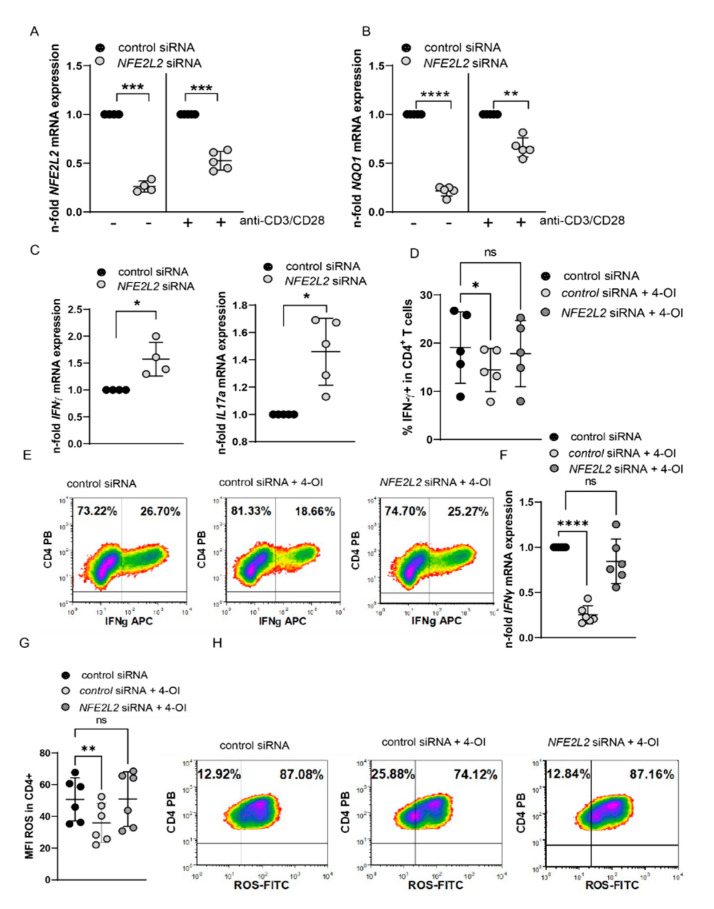
4-OI regulates T cells by NRF2. (**A**–**D**) PBMCs were transfected with NFE2L2 siRNA (10 nM) and control siRNA using Neon transfection system. After incubation for 24 h, cells were left either unstimulated or stimulated with anti-CD3/CD28 for 24 h. (**A**) NFE2L2 mRNA expression in unstimulated (N = 4 for donors and three individual experiments; SD: 0.056655) and stimulated cells (N = 5 for donors and four individual experiments; SD: 0.09612), a one sample *t*-test. (**B**) NQO1 mRNA expression in unstimulated (N = 5 for donors and four individual experiments; SD: 0.05095) and stimulated cells (N = 5 for donors and four individual experiments; SD: 0.09892), a one sample *t*-test. (**C**) IFN-γ mRNA expression in stimulated cells (N = 4 for donors and three individual experiments; SD: 0.3139) and IL17a mRNA expression in stimulated cells (N = 5 for donors and four individual experiments; SD: 0.2456), a one sample *t*-test. (**D**–**G**) CD4+ T cells were transfected with NFE2L2 siRNA (10 nM) and control siRNA using the Neon transfection system. After incubation for 24 h, cells were left either unstimulated or stimulated with anti-CD3/CD28 for 48 h in the presence or absence of 20 µM 4-OI. (**D**) IFN-gamma percentages in CD4+ T cells (N = 6 for donors and three individual experiments). (**E**) Representative density plots showing percentages of IFN-γ+ T cells, a RM-One way ANOVA, and Dunnett’s multiple comparisons test were performed. (**F**) Statistical analysis of IFN-γ expression (N = 6 for donors and three individual experiments; SD: 0.1004 & 0.2462), as assessed by flow cytometry, a one sample *t*-test was performed. (**G**) Statistical analysis of MFI of ROS in CD4+ T cells (N = 6 for donors and three experiments), an RM-One way ANOVA, and Šidák’s multiple comparisons test was performed. (**H**) Representative density plots of (**G**). Stastical significance represented as * *p* < 0.05, ** *p* < 0.01, *** *p* < 0.001, **** *p* < 0.0001 and ns is not significant.

4-OI affects other pathways beyond NRF2. We, therefore, investigated whether 4-OI-mediated effects are indeed NRF2 dependent. To this end, we established knock-down of NRF2 in primary T cells by siRNA transfection and managed to achieve a downregulation of NRF2 and NQO1 in cells that were transfected with NFE2L2 siRNA compared to transfections with a control siRNA (Figure 6A,B, Supplementary Figures S2 and S3). Furthermore, NRF2 silencing enhanced IFN-γ and IL-17a mRNA expression (Figure 6C), which mimics the phenotype of SF T cells characterized by low NRF2 but high IL17 and IFN-γ expression. Using this approach, we were able to demonstrate that 4-OI mediated downregulation of IFN-γ required NRF2, as NFE2L2 siRNA transfected cells did not show significant downregulation of IFN-γ producing T cells or IFN-γ mRNA expression after 4-OI treatment (Figure 6D–F). In addition, reduced ROS expression after 4-OI treatment also required sufficient NRF2 expression (Figure 6G). These experiments provide evidence that 4-OI effects are NRF2-dependent and Itaconate/NRF2 axis regulates ROS and IFN-γ production in T cells.

## 4. Discussion

Our study demonstrates that the redox metabolism of T cells within the local inflammatory environment of JIA patients is strongly connected with fatty acid metabolism. Inflamed tissues are characterized by reduced glucose and glutamine levels but high availability of fatty acids. Consequently, the usage of fatty acids to maintain the cell’s metabolic needs might be mandatory for the survival and function of T cells in inflamed tissues. Recently, Hradilkova et al. showed that PD-1+ T cells within the inflamed joints of JIA patients are regulated by TWIST1 and adapt their metabolism to become entirely dependent on fatty acid oxidation [41] Moreover, TWIST1 protected them from oxidative stress. Our findings also provide further insights into the T cellular redox metabolism during chronic inflammation. In detail, we show that despite enhanced ROS production, NRF2 is downregulated in SF T cells and that this correlates with fatty acid metabolism. Consequently, the upregulation of NRF2 reversed some of the cells’ inflammatory properties. The observed downregulation of NRF2 in these highly inflammatory T cells was an unexpected finding, as NRF2 expression has been shown to increase in activated murine and human T cells [16,17,18]. As NRF2 downregulation can also be induced by the incubation of HC CD4+ T cells with SF, we suggest that components of the SF suppress NRF2 activation. SF T cells showed signs of greatly enhanced metabolism with raised glucose and fatty acid uptake and a high content of lipid droplets. Accumulation of intracellular lipid droplets in autoreactive T cells has been shown in RA, psoriasis and SLE as well [42]. Intracellular lipids play a significant role during effector T cell differentiation and during clonal expansion when massive cellular proliferation places high biosynthetic demands on the cells [43]. The NRF2 activator 4-OI downregulated fatty acid uptake, ROS production and expression of IFN-γ in T cells in an NRF2 dependent manner. 4-OI is a cell-permeable derivative of itaconate [44]. Until now most itaconate studies have been performed in macrophages, where itaconate was found to be produced in response to LPS stimulation. Beyond its effects on Nrf2 activation, itaconate directly inhibits the enzymatic activity of SDH, resulting in a blockade of succinate-mediated inflammatory processes [13,14,15]. Our study highlights an expanded role of 4-OI, critical to T cells in inflammation. Our data are in line with those of Nastasi et al., who describe a downregulation of IFN-γ and T cell proliferation in 4-OI but not in itaconate-treated T cells. These authors suggest that the effects of 4-OI on human CD4+ T cells are also mediated through activation of NRF2, as they observed increased expression of NRF2 target genes following 4-OI treatment and only a marginal increase in intracellular succinate accumulation [45].

The limitations of our study begin with the low number of samples. In addition to this, low cell numbers within the synovial fluid hindered us to use seahorse assays or other more comprehensive techniques to interrogate cellular metabolism. Further studies should also include samples of RA and test other chronic inflammatory conditions. Other limitations include the lack of any in vivo testing of 4-OI effects. A murine arthritis model with exogenous 4-OI treatment is planned to confirm the functional relevance of our findings in vivo. In a recent paper, the exogenous supplementation of nasal itaconate derivatives was shown to diminish the manifestation of house dust mite type 2 airway inflammation in a murine asthma model [46]. The authors suggest that this was related to the effects of itaconate in DCs. However, they did not investigate any direct effect of 4-OI on T cells [46]. In a vaccination model with an F. tularensis live vaccine strain, itaconate-deficient mice developed a prolonged primary infection but were more resistant to secondary infection with virulent F. tularensis relative to wild-type controls. Improved resistance to the secondary challenge was associated with both increased numbers and effector function of CD4+ and CD8+ T cells in itaconate-deficient mice. However, additional data suggest that improved T cell responses were not T cell intrinsic [47]. By inhibiting inflammatory cytokine production in human macrophages, the study by Tang et al. obtained findings pointing to a beneficial role of 4-OI in SLE [48]. In addition, itaconate and its derivates show anti-inflammatory effects in several preclinical models of inflammation such as sepsis and psoriasis [49]. Furthermore, global NRF2 activation appears to be beneficial in certain autoimmune diseases. Dimethyl fumarate (DMF) is an NRF2 activator and has been approved for the treatment of relapsing-remitting multiple sclerosis and psoriasis [50]. In conclusion, several studies demonstrate the anti-inflammatory effects of 4-OI and NRF2 activation in various immune cells. Nevertheless, through its involvement in NRF2 activation and redox metabolism, 4-OI may also cause unwanted side effects. In-depth studies will be needed to establish whether 4-OI-induced NRF2 activation can be used effectively in the treatment of JIA.

## 5. Conclusions

NRF2 could be a potential regulator in CD4+ T cells during chronic inflammation and could instigate a drift toward disease progression or regression, depending on the inflammatory environment.

## Figures and Tables

**Table 1:** Details of oligoarticular JIA patients with treatment provided.

Gender	Age (Years)	Treatment
Female	4	none
Female	15	none
Male	11	none
Female	7	none
Male	14	none
Female	3	none
Female	11	none
Male	4	none
Male	14	none
Female	8	Methotrexate
Female	13	none
Male	16	Methotrexate & Decortin
Female	10	none
Male	6	none
Male	12	none
Female	6	Ibuprofen
Female	12	none
Female	6	none

## Data Availability

Data are available upon reasonable request to the corresponding author. All relevant data generated during this study are included in the article.

## Supplementary Materials

The following supporting information can be downloaded at: https://www.mdpi.com/article/10.3390/antiox11122426/s1, Figure S1: Gating strategies. Represent5ative Dot Plots and histograms showing gating strategies and calculation of delta MFI. Figure S2: NRF2 protein is reduced in with SF incubation. For analysis of NRF2 protein expression by Western blot, PBMCs were stimulated for 4 hours with10 % HC (N = 6 for donors and 2 individual experiments) or 10 % SF (n = 8 for donors and 2 individual experiments). Afterwards the nuclear protein fractions were extracted, and Western blots performed with antibodies against NRF2 and Lamin A/C. Lamin A/C was used as a loading control.Quantification of NRF2 protein expression normalized to Lamin A/C protein expression; a Mann-Whitney test was used to test for significance; Figure S3: NRF2 protein is downregulated after siRNA transfection. For analysis of NRF2 protein expression by Western blot, PBMCs were transfected with control siRNA or NFE2L2 siRNA. After 22 hours the nuclear protein fractions were extracted, and Western blots performed with antibodies against NRF2 and Lamin A/C. Lamin A/C was used as a loading control. Figure S4: DeltaCT levels of NFE2L2 and NQO1 expression after siRNA transfections. PBMCs were transfected with NFE2L2 siRNA (10nM) and control siRNA using neon transfection system. After incubation for 24 hours, cells were left either unstimulated or stimulated with anti CD3/CD28 for 24 hours. A) NFE2L2 mRNA expression in unstimulated (N = 4) and stimulated (N = 5). B) NQO1 mRNA expression in unstimulated (N = 5) and stimulated cells (N = 5).
