# Supplementary material for: NRF2/Itaconate Axis Regulates Metabolism and Inflammatory Properties of T Cells in Children with JIA
Source: Antioxidants (Basel). 2022 Dec 8;11(12):2426. doi: 10.3390/antiox11122426 (PMC9774972; doi:10.3390/antiox11122426)
Supplement: Supplementary file 1 [file antioxidants-11-02426-s001.zip › antioxidants-2084129-supplementary.pdf]

Supplementary files:

ROS

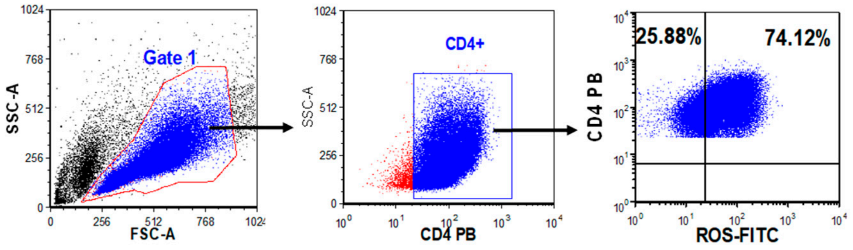

IFN-γ

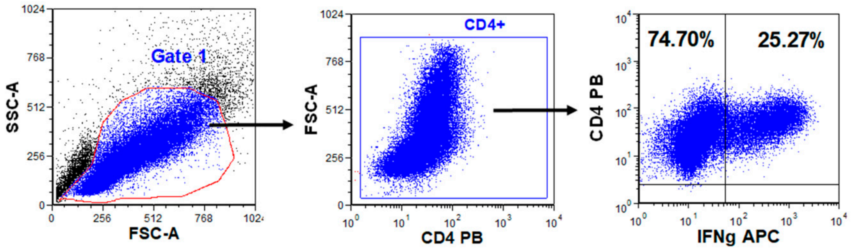

cell proliferation dye

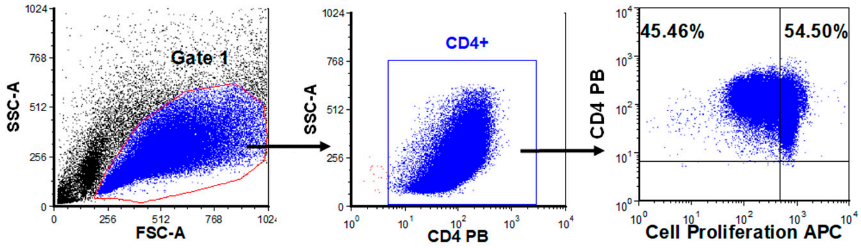

Mitotracker & Mitosox assay

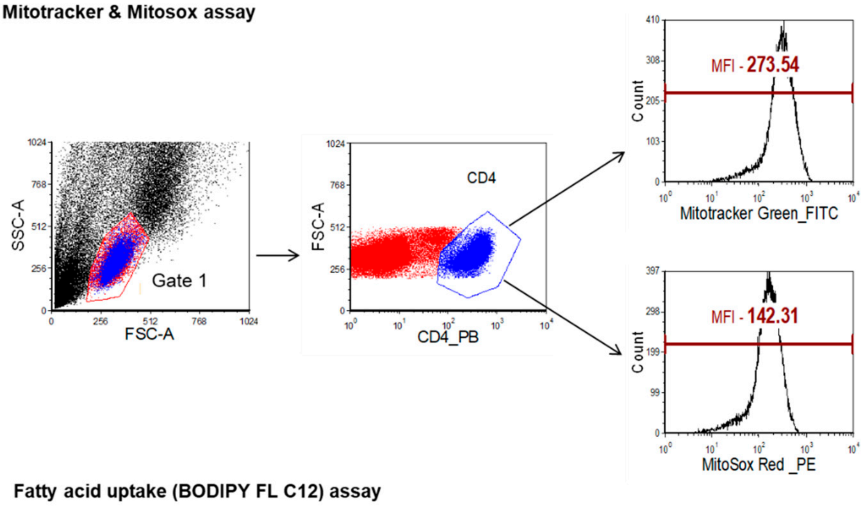

Fatty acid uptake (BODIPY FL C12) assay

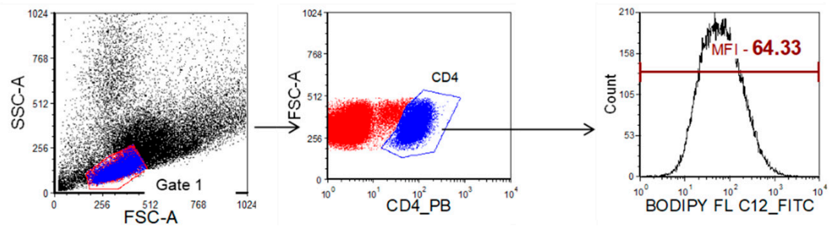

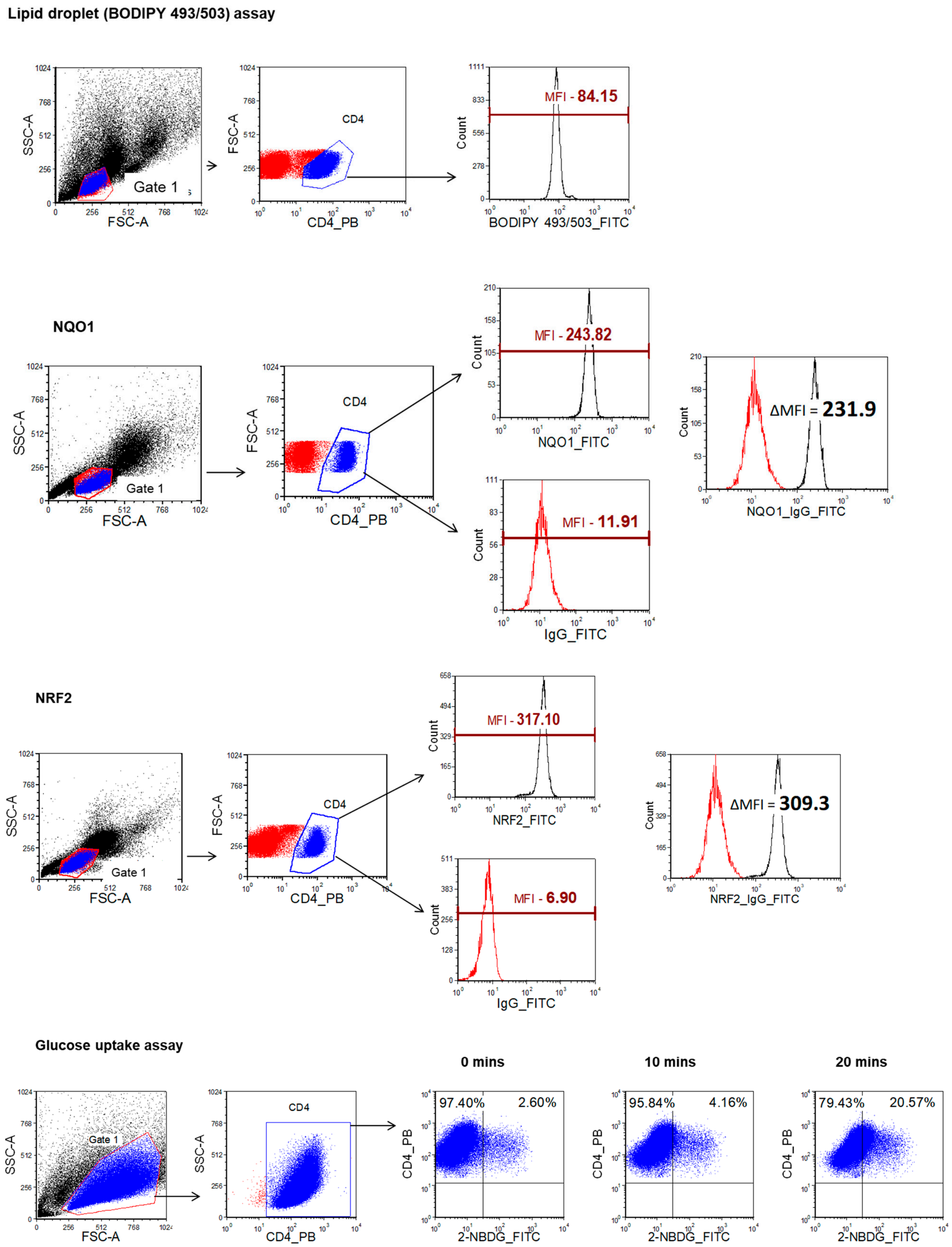

Figure S1: Gating strategies. Representative Dot Plots and histograms showing gating strategies and calculation of ∆ MFI.

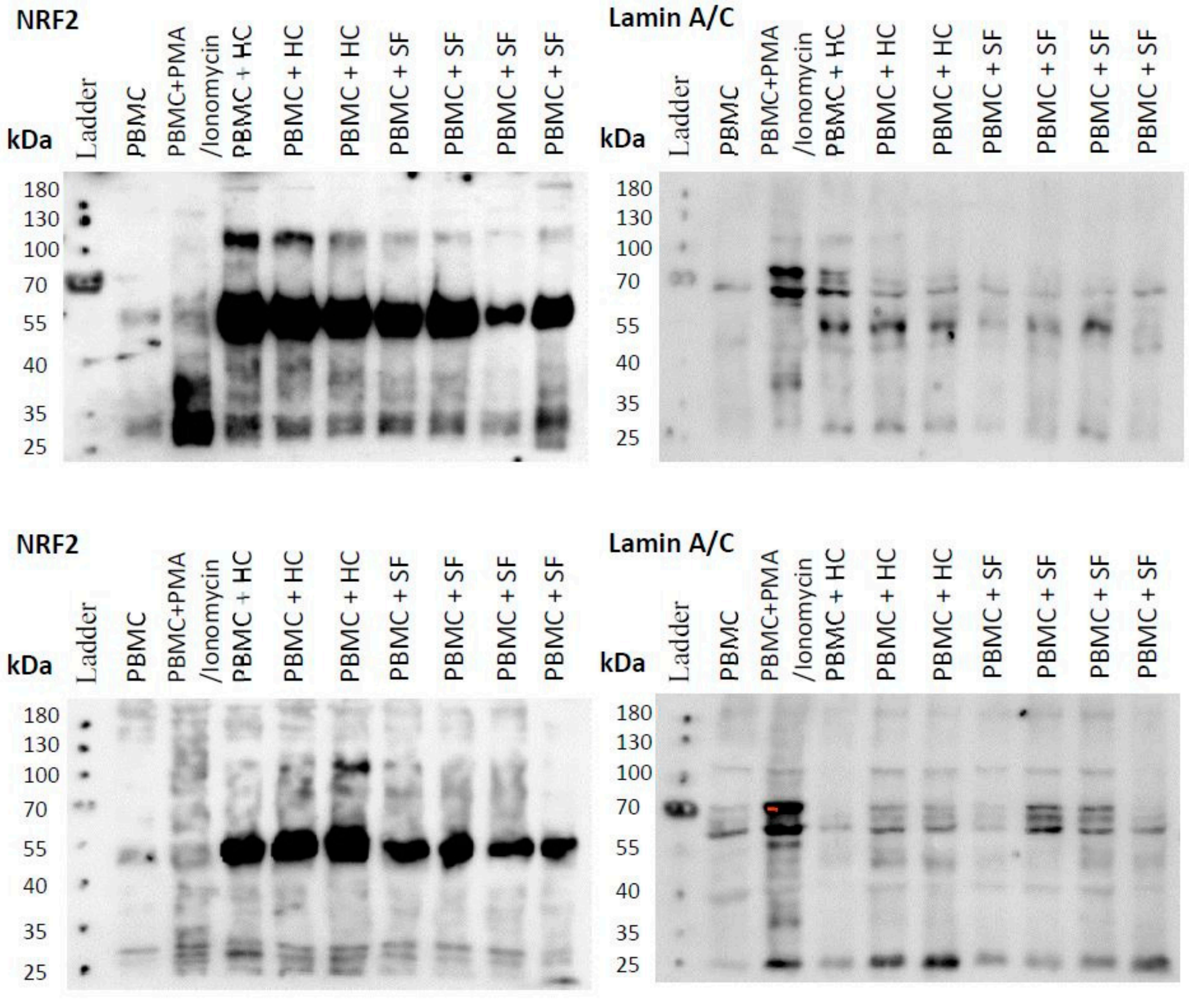

Figure S2: NRF2 protein is reduced with SF incubation. For analysis of NRF2 protein expression by Western blot, **A)** PBMCs were stimulated for 4 hours with10 % HC (N = 6 for donors and 2 individual experiments) or 10 % SF (n = 8 for donors and 2 individual experiments). Afterwards the nuclear protein fractions were extracted, and Western blots performed with antibodies against NRF2 and Lamin A/C. Lamin A/C was used as a loading control. Quantification of NRF2 protein expression normalized to Lamin A/C protein expression.

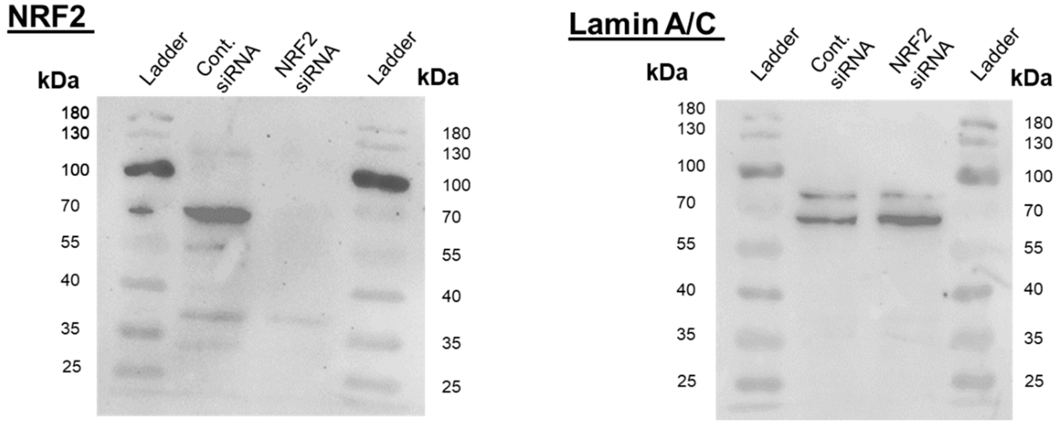

Figure S3: NRF2 protein is downregulated after siRNA transfection. For analysis of NRF2 protein expression by Western blot, PBMCs were transfected with control siRNA or *NFE2L2* siRNA. After 22 hours the nuclear protein fractions were extracted, and Western blots performed with antibodies against NRF2 and Lamin A/C. Lamin A/C was used as a loading control.

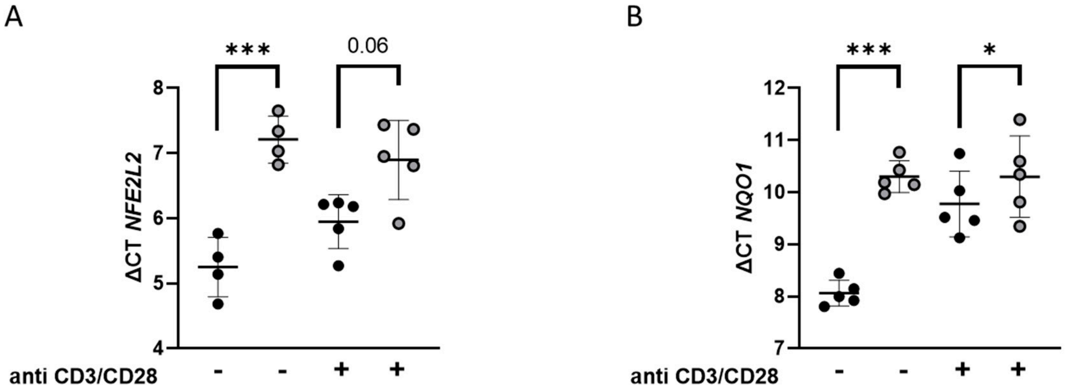

Figure S4: ΔCt levels of *NFE2L2* and *NQO1* expression after siRNA transfections. PBMCs were transfected with *NFE2L2* siRNA (10nM) and control siRNA using Neon transfection system. After incubation for 24 hours, cells were left either unstimulated or stimulated with anti-CD3/CD28 for 24 hours. A) *NFE2L2* mRNA expression in unstimulated (N=4) and stimulated cells (N=5). B) NQO1 mRNA expression in unstimulated (N=5) and stimulated cells (N=5).
